# Brexit and Animal Welfare Impact Assessment: Analysis of the Threats Brexit Poses to Animal Protection in the UK, EU and Internationally

**DOI:** 10.3390/ani9030117

**Published:** 2019-03-26

**Authors:** Steven P. McCulloch

**Affiliations:** Department of Politics and Society, University of Winchester, Winchester SO22 4NR, UK; Steven.McCulloch@winchester.ac.uk; Tel.: +44-(0)1962-827-465

**Keywords:** animal health, animal welfare, animal welfare impact assessment, animal protection policy, Brexit, Common Agricultural Policy, Conservative Party, European Union, Labour Party, World Trade Organisation

## Abstract

**Simple Summary:**

The British people voted in a 2016 referendum to leave the European Union and the United Kingdom (UK) is set to leave the EU in 2019. Brexit is a major political change and it presents both threats and opportunities for animal protection. This paper assesses the threats that Brexit poses to animal protection in terms of five criteria. These are first, the political situation; second, regulatory changes; third, economic and trade factors; fourth, institutional considerations; and fifth, EU and international impacts. The EU has the most progressive animal welfare laws in the world. The UK Conservative Government, which is delivering Brexit, has a mixed record on animal protection. Brexit is forecast to have a negative impact on the UK economy, which is likely to negatively affect animal welfare. A major threat of Brexit is the import of meat and dairy products to the UK raised in lower welfare standards from nations such as the United States (US). The development of Brexit policy suggests there is a significant risk that this threat will materialise. Furthermore, Brexit will result in a reduced political lobby within the EU for progressive animal protection reform. Despite the UK being a progressive animal protection nation, she will have less power to exert this influence to improve animal welfare outside of the EU. Brexit poses substantial risks to weaken animal protection in the UK, EU and internationally. Further research is needed to assess the opportunities presented by Brexit to judge whether Brexit will be overall positive or negative for animal protection.

**Abstract:**

The British people voted in a 2016 referendum to leave the European Union (EU). Brexit presents both threats and opportunities to animal protection in the United Kingdom (UK), EU and internationally. This paper discusses threats to animal protection in terms of five criteria. These are first, political context; second, regulatory changes; third, economic and trade factors; fourth, institutional and capacity-related factors; and fifth, EU and international considerations. The EU has the most progressive animal welfare laws in the world. The Conservative Government delivering Brexit has a mixed record on animal protection. Major time and resource constraints inherent in Brexit risk negatively impacting animal protection. Brexit is projected to have a negative economic impact, which is generally associated with lower animal welfare standards. The development of Brexit policy suggests there to be a substantial risk that the major threat of importing lower welfare products to the UK will materialise. Brexit will reduce the political influence of the progressive animal protection lobby in the EU. Post-Brexit, the politically and economically weakened EU and UK risks a detrimental impact on animal protection on an international scale. Brexit poses substantial threats to animal protection, with a high risk that many threats will materialise. Further research is needed to assess the opportunities presented by Brexit to judge whether Brexit will be overall positive or negative for animal protection.

## 1. Introduction

The British people voted in a 2016 referendum to leave the European Union (EU). The United Kingdom (UK) has been a member of the EU since the Maastricht Treaty was signed in 1993 and before that a member of the European Communities (EC) since 1973. EU regulation has had a major influence on UK animal health and welfare policy. Similarly, as one of the larger members of the EU, and with a history as a regional and global leader in animal welfare, the UK has had a substantial influence on EU animal protection policy [1,2,3]. For instance, in the 1990s the UK Government lobbied the EU to recognise the sentience of animals. This resulted in a protocol on animal welfare in the Treaty of Amsterdam in 1997, which was later strengthened to Article 13 of the Treaty of Lisbon [4]. Furthermore, the UK has been instrumental in reforms across the EU including the prohibition of veal crates (2007), barren battery cages for hens (2012) and the regulation of sow stalls (2013) [1].

Brexit is a highly controversial political issue in the UK that will have a major impact on British governance and policy. The vote to leave was won by a narrow margin of 51.9% to 48.1%. The referendum has divided families, constituencies, Parliament and even nations within the UK [5,6]. The leader of the Conservative Party and Prime Minister of the UK, Theresa May, has committed the Government to implementing Brexit. The UK gave notice of Article 50 in March 2017 and was to leave the EU by the end of March 2019 [7]. This would be followed by an implementation or transitional period from Brexit day until 31 December 2020. During the implementation period, the UK will leave the EU but remain in the single market and customs union. The implementation period means that the free flow of goods, capital and services continues until 2021 [8,9].

The Government has committed to leaving the EU single market and customs union after the implementation period. However, Government policy is for the UK to have a common rulebook in agricultural goods, effectively remaining in the single market for this sector [10,11]. Despite this, there has been strong opposition to the Government’s policy in Parliament, led by the Eurosceptic European Research Group (ERG) of the Conservative Party. The ERG favours a hard Brexit with a complete rupture of the UK from the EU and a Canada-style trade agreement with the EU [12,13].

The UK Government’s Withdrawal Agreement and Political Declaration was signed off by the EU Council on 25 November 2018. The Withdrawal Agreement is an international agreement between the UK and EU setting out the terms of the UK’s withdrawal from the EU [14]. The Political Declaration sets out the scope of the future relationship between the UK and the EU [15]. However, the UK Parliament overwhelmingly rejected Theresa May’s Withdrawal Agreement by 432 to 202 votes on 15 January 2019 [16]. Jeremy Corbyn, the leader of the opposition Labour Party, tabled a vote of no confidence in the Prime Minister the following day. However, Theresa May, with the support of Northern Ireland’s Democratic Unionist Party (DUP) confidence and supply partners, won the vote by a narrow margin of 19 votes [17].

At the time of writing in March 2019 the political situation remains extremely volatile. Parliament has rejected Theresa May’s Brexit deal. However, there appears to be no majority support for any version of Brexit meaning that Parliament is gridlocked [18]. If the UK does not agree a divorce deal with the EU it will crash out of the EU in a no-deal Brexit. Further options include Theresa May renegotiating with the EU or calling a General Election to seek a mandate to deliver her Brexit. Both of these options would mean that the UK Government would have to ask the EU to extend Article 50 and to provide the necessary time to implement Brexit. Some MPs argue that since Parliament is gridlocked, and because we now know a lot more about the implications of Brexit, there should be a second referendum both to see if the public still wants to leave the EU and, if it does, what type of Brexit it prefers [19].

Theresa May has negotiated further with the EU, who have advised they are not able to reopen the Withdrawal Agreement. The Prime Minister responded by scheduling a series of three votes in Parliament. A second meaningful vote on Theresa May’s deal on March 12 2019 was lost by a margin of 149 votes. On March 13, Parliament rejected leaving the EU without a deal under any circumstances by a narrow margin of 312 to 308. Finally, on 14 March 2019, Parliament voted by a majority of 211 votes to seek an extension to Article 50 and delay Brexit. Given this series of votes, there are several potential scenarios. These include a further vote on the Prime Minister’s deal, renegotiation with the EU, a general election, a vote of no confidence in the Prime Minister, a second referendum on EU membership, or revoking Article 50 and abandoning Brexit altogether [20].

Several reports have been published on the impact of Brexit on animal protection. Brexit represents a huge political change for UK governance and the reports describe the various threats and opportunities that Brexit poses. They provide an indispensible resource on the potential impacts of Brexit on animal welfare. This paper seeks to go beyond a broad discussion of the threats and opportunities by assessing whether, all things considered, Brexit will be positive or negative for animal protection. The paper builds on discussion in McCulloch [3], which focuses on the legal and political context of Brexit and animal protection. This paper is structured in two parts. The first part of the paper describes a framework to assess the threats and opportunities that Brexit presents for animal protection. The second part of the paper assesses the threats that Brexit poses to animal protection.

## 2. A Framework to Investigate the Impact of Brexit on Animal Welfare

Brexit has the potential to have major impacts on animal protection in the UK, the EU and internationally. However, how Brexit will actually impact animal protection is highly complex. There are various forms of Brexit, and different models of the post-Brexit UK-EU relationship that might materialise. Each of these will have different impacts on animal protection. There are different categories of animals, including farmed, wild, experimental and companion, that will be affected by Brexit. Furthermore, there is a multitude of uncertainties, for instance how key players in the UK, EU and future trading nations will act, which will impact animal welfare. McCulloch [3] has described a framework to investigate the impact of Brexit on animal protection. The framework is reproduced below.

1. What is the current relationship between the UK and the EU in animal health and welfare policy, i.e., what is the status quo?
2. What is the political context of Brexit, i.e., what are the political considerations that are likely to determine the impact of Brexit on animal protection?
3. What are the threats and opportunities to animal protection of Brexit?
4. What are the threats and opportunities to animal protection of Brexit to different categories of animals?
5. What are the threats and opportunities to animal protection of different forms of Brexit?
6. What are the threats and opportunities to animal protection of Brexit geographically, i.e., in the UK, the EU and internationally?
7. What are the magnitudes of the various threats and opportunities of Brexit?
8. How likely are the various threats and opportunities of Brexit to animal protection to materialise?
9. All things considered, will Brexit be a net positive or negative for animal protection in the UK, EU and internationally?

McCulloch [3] has discussed questions 1–5 of this framework. That paper discusses how EU and UK animal protection regulation has co-evolved. Around 80% of all UK animal protection regulation is based on EU laws [21]. The political context of Brexit has also been discussed, since this will have a very significant impact on how Brexit impacts animal protection. It is the decisions and actions of political representatives in the UK, the EU and internationally that will have a major impact on animal protection. McCulloch [3] discussed soft and hard forms of Brexit, as well as trade relations based on World Trade Organisation (WTO) rules.

In McCulloch [3], it was shown that whether Brexit has an overall positive or negative impact on animals will be determined by how Brexit impacts farmed animals. This claim is based on two premises. First, there are far larger numbers of farmed animals compared to those in other categories. One billion land farmed animals are raised and slaughtered annually in the UK [22], which compares for instance to four million experimental procedures conducted on animals [23]. Secondly, membership of the EU means that UK agricultural policy has been determined by the Common Agricultural Policy (CAP) since 1973. Brexit is therefore likely to mean far more significant regulatory and policy changes for farm animal regulation compared to other categories of animals. 

Finally, McCulloch [3] claimed that a major threat to animal welfare posed by Brexit is the import of lower welfare agricultural products from nations such as the United States (US). The greatest opportunity Brexit presents is to reform UK agricultural policy based on rewarding high welfare as a public good. A soft Brexit means closer alignment between the EU and UK. For instance, a soft Brexit means that the UK remains part of the EU single market and/or customs union or policy to that effect that applies to agricultural products. A hard Brexit, in contrast, means the UK will leave both the single market and customs union. McCulloch [3] claimed that the major threat of importing lower welfare products is more likely with a hard rather than a soft Brexit. For instance, a hard Brexit and trade based on WTO terms has been described as ‘catastrophic’ for animal welfare [24]. The discussion in the remainder of this paper focuses on questions 6–9 in the framework above.

## 3. Assessment of the Threats to Animal Welfare

How Brexit affects animal protection will be determined by a number of complex and interrelated factors. Some of these will be under the control of the UK Government; others will be influenced by the EU and future non-EU trade partners. First, Brexit will be influenced by whether the UK concludes a soft or a hard Brexit. Secondly, the political nature of the UK Government and Parliament will have a substantial impact on how Brexit affects animals. Thirdly, the impact of Brexit on animals will be influenced significantly by the EU. These impacts will be on animals in the UK, EU and internationally. Fourthly, non-EU countries such as the US may play a key role in how Brexit affects animal protection. Finally, there are a number of uncertainties such as how WTO rules will be interpreted for trade restrictions based on animal welfare grounds.

Table 1 summarises the major threats that Brexit poses to animal protection. The key threats are categorised as political, regulatory, economic and trade, institutional- and capacity-related, and EU and international factors. The order is intended to be logical, beginning with the broader political context of Brexit, moving to regulatory changes, through to economic and trade considerations, and then institutional and capacity-related factors. The final category in the table moves to the discussion of how Brexit might impact EU and international animal protection. The discussion of these threats in the text follows the same order as that presented in Table 1.

## 4. Political Factors

### 4.1. The European Union Has the Most Progressive Animal Welfare Laws in the World

The EU has the most progressive animal welfare laws in the world. Article 13 of the Treaty of Lisbon recognises animals as sentient beings and mandates that member states pay full regard to animal welfare when formulating and implementing policy. In the farmed animal context, the EU has prohibited veal crates and barren battery cages and limits the use of sow stalls. Furthermore, the EU has banned the testing of cosmetics and ingredients on animals. In the World Animal Protection (WAP) Animal Protection Index, EU nations score highly; with the UK and Austria scoring ‘A’ and Germany, the Netherlands, Denmark and Sweden ‘B’ [25]. EU animal health and welfare regulation is discussed further in McCulloch [3]. 

Given that the EU has the most progressive animal welfare regulation in the world, it seems prima facie problematic to claim that leaving the EU will benefit animal protection. To elaborate, consider Brexit in a geopolitical and trade context. The United Kingdom of Great Britain and Northern Ireland is a set of islands off mainland Europe. It has strong links to the EU as a member state and to the US as a traditional political ally and trading partner [26]. Brexit means that the UK leaves the political institutions of the EU. If the UK leaves the single market and customs union, Brexit also means a looser trading relationship with the EU. Leaving the customs union in particular means that the UK is able to determine its own trade policy. Those who support Brexit argue that leaving the EU can increase opportunities to trade with nations such as the US. For instance, the Secretary for International Trade Liam Fox has stated the following:

After we leave the EU on March 29, the UK will have an independent trade policy covering goods and services, we will be able to set our own tariffs, and we will be able to negotiate, sign and ratify new free trade agreements. And among our first priorities is an agreement with the United States.

[27]

A move away from the EU and toward the US, for instance, may be a major problem for animal protection. Whereas the EU has very high welfare standards relative to other nations, the US has some of the least progressive legislation in the developed world [28]. Furthermore, the US is far more economically and politically powerful than the UK. The power imbalance between the UK and US will influence trade negotiations post-Brexit. Indeed, this issue has already begun to unfold during President Trump’s state visit to the UK in summer 2018. Trump criticised Theresa May’s Chequers Agreement, which was likely motivated in part by the President wanting to open up the UK market to US beef and other exports [29]. 

In March 2019, the US Government published its objectives for a post-Brexit US–UK trade deal. The US document includes the negotiating objective to secure ‘comprehensive market access for U.S. agricultural goods in the UK by reducing or eliminating tariffs’. The US negotiating stance further aims to ‘Eliminate practices that unfairly decrease U.S. market access’ including ‘non-tariff barriers that discriminate against U.S. agricultural goods’ (pp. 2–3) [30].

On the same day as the US Government published its negotiating objectives, the US ambassador to the UK, Robert Wood Johnson, wrote an article in the *Daily Telegraph* [31,32]. Johnson urged the UK to embrace US farming methods. In his article, Johnson hailed US practices such as chlorine washing chicken and hormone implants for cattle as the ‘future of farming’ while the EU was a ‘museum of agriculture’ and stuck in the past. In response to the publication of the US Government document, the UK Government has reiterated that it will not lower food safety standards after Brexit [33].

### 4.2. Inherent Threats in Massive Political Change

Brexit constitutes a massive political upheaval for the UK. The UK has been a member of the EU since the Maastricht Treaty was signed in 1993, and before that a member of the European Communities (EC) since 1973. Around 80% of UK animal protection law derives from the EU [21]. Much of the threat in this context has been neutralised through the EU Withdrawal Act. The EU Withdrawal Act 2017 nationalised EU legislation, particularly regulations, into UK law. Despite this, the Act gives ministers substantial powers to amend legislation without the involvement of Parliament [34,35].

A very good example of a threat that arises from massive political change is Government policy recognising animal sentience and associated duties. The UK Government has obstructed the only piece of animal protection regulation not carried over by the EU Withdrawal Act. Article 13 of the Treaty on the Functioning of the European Union (TFEU) states that animals are sentient beings and confers a duty on the European Commission and member states to pay full regard to animal welfare when formulating and implementing policy [36]. Article 13 is not carried over to UK law in the EU Withdrawal Act 2018 because it is part of the TFEU. The TFEU is about the power and competence to legislate under EU law so Article 13 would not be meaningful if directly carried over to UK law. The loss of Article 13 prompted Caroline Lucas, the sole Green Party Member of Parliament (MP), to table an amendment to incorporate the provisions of Article 13 into the EU Withdrawal Bill, in order to maintain the same degree of animal protection in the UK post-Brexit [37].

Conservatives MPs were whipped to vote against the amendment, and the Government won the vote by 313 votes to 295. (Government whips are party officials that enforce party discipline by providing voting instructions to Parliamentarians.) The Government argued that transposing Article 13 TFEU was unnecessary as recognition of animal sentience is implicit in the Animal Welfare Act 2006 [38]. The Conservative Government’s position on the animal sentience issue led to a media furore that was highly critical of the Government [39]. The Government’s position was roundly criticised by animal protection non-governmental organisations (NGOs), who argued that animals would lose a significant degree of protection. They pointed out that the Animal Welfare Act, for instance, does not cover wild animals, whilst Article 13 covers all sentient animals [40]. 

In response to continued media attention and public disquiet, the Government published its Animal Welfare (Sentencing and Recognition of Sentience) Bill. Clause I of the Bill recognised animals as sentient and Clause II of the Bill increased the maximum punishment for animal cruelty from six months to five years. However, Clause I of the draft Bill significantly watered down the provisions in Article 13. The drafting of the Bill included ‘regard’ and not the ‘full regard’ of Article 13 and consulted on the level of regard. Furthermore, the duty to pay regard was now limited to ministers of the state, when in Article 13 it had been conferred on member states generally. Given that local government has a substantial role especially in the implementation of animal protection policy, limiting the duty to government ministers thus reduces the scope of the duty. Thus, the draft Animal Welfare (Sentencing and Recognition of Sentience) Bill both reduced the degree of the duty, as well as the scope of responsibility [37,41]. 

The watering down of Article 13 post-Brexit constitutes a downgrade in the moral and legal status of animals [42]. The Bill was heavily criticised by the Environment, Food and Rural Affairs Committee (EFRA Comm), the Parliamentary body that scrutinises Department for Environment, Food and Rural Affairs (Defra) policy. EFRA Comm recommended that the Government split the Bill into its two clauses, pass Clause II of the Bill, and re-draft and consult further on Clause I of the Bill that included provisions related to sentience. The Government has followed this advice, and has announced it will publish a re-drafted Bill on sentience. Arguably, the current situation means there is a real risk that the UK will not have an Act in place to recognise animal sentience and confer the relevant duties on Government during the implementation period and immediate post-Brexit phase. During this time, the Government will be under significant pressure when negotiating trade deals involving agricultural products with the EU and third countries such as the US [43]. If a Bill recognising sentience and conferring an obligation on the UK Government were enacted prior to negotiating trade deals, ministers would need to respect the duty, for instance to pay regard to animal welfare. Without such an obligation in place, the UK Government would have no such obligation to pay regard to the welfare of sentient animals. Given that Brexit may lead to the UK negotiating hundreds of trade deals around the world, and the potential impact of such trade deals on billions of animals going forward, having sentience legislation in place during such a time is enormously important for animal protection.

Based on such concerns about Brexit and in particular recognition of sentience, a coalition of 36 of the largest UK-based animal protection organisations organised a Better Deal for Animals campaign in February 2019. The coalition, including the Royal Society for the Prevention of Cruelty to Animals (RSPCA), Compassion in World Farming (CIWF) and Humane Society International (HIS) is lobbying for an Animal Welfare Advisory Council to provide robust independent advice to government departments. The coalition argues that the Council should include experts in animal welfare and ethics and carry out prospective animal welfare impact assessments to inform Government policy [44,45].

In a YouGov poll commissioned by the coalition, 81 per cent of respondents preferred animal welfare laws in the UK to be maintained or made more extensive after Brexit. However, 56 per cent were not confident that the UK Government would keep its commitments to recognise animal sentience and strengthen animal protection after Brexit. Two thirds of respondents supported the establishment of an Animal Protection Committee to provide expert independent advice to Government. In terms of future trade, 80 per cent supported the inclusion of a clear requirement that imported animal products meet or exceed British animal welfare standards [46,47].

### 4.3. Brexit Is Motivated and Delivered by the Political Right

The academic animal welfare literature in the UK has been dominated by animal welfare science since the publication of the Brambell report in 1965 [48]. With some notable exceptions [41,49,50], there are few authors that have written about animal protection in a political science context. This is unfortunate because developments in animal welfare science require changes at the political level for meaningful reform. Furthermore, Brexit represents political change of such a magnitude that it has the potential for substantial and long-term impacts on animals. The impact that Brexit ultimately has on animal protection will relate directly to decisions make by key actors in Westminster, Whitehall and Brussels.

Elsewhere, I have discussed the political context of Brexit [3]. Britain has had an ambivalent relationship with Europe since it joined the EC in 1973. In the 1970s of the two major British parties it was Labour that was the more divided on Europe. During the 1975 referendum campaign to remain in the EC, the Prime Minister Harold Wilson permitted his Eurosceptic ministers, including Tony Benn, to campaign to leave the EC. In contrast, leading up to 1975 the Conservative Party had consistently supported membership of the EC [51].

In the 1980s, however, the attitudes of the two major parties toward Europe began to shift. Margaret Thatcher was increasingly concerned about European integration and her 1988 Bruges speech resulted in a generation of Conservative Eurosceptic MPs. In the 1990s the Conservative Party was increasingly split on the EU under John Major [52]. The United Kingdom Independence Party (UKIP) was founded in 1993 and it was the later leakage of Conservative voters to UKIP [53] that prompted the leader of the Conservatives and Prime Minister David Cameron to pledge to hold an in/out referendum on the EU if his party was elected to Government in the 2015 general election [54].

In the 1990s, the Labour Party under Tony Blair became more pro-European. Today’s Labour Party membership and its MPs are both pro-European [55]. However, the Labour leader Jeremy Corbyn, influenced by Benn in the 1970s, is more Eurosceptic than his party, although he campaigned in the 2016 referendum to remain in the EU [56]. Thus, despite Euroscepticism being more associated with the right wing of the Conservative Party in the UK today, there are elements of Euroscepticism in the Labour Party, and especially its leadership.

The motivation for the EU referendum and subsequently Brexit has been described above. Theresa May has appointed to her Cabinet high-profile right-wing Eurosceptics to deliver Brexit such as David Davies (Department for Exiting the EU), Liam Fox (Department for International Trade) and Boris Johnson (Foreign Office) [3]. The Prime Minister has been accused of pandering to the ERG, formed by the Eurosceptic right wing of her party, during the Brexit negotiations [57,58]. The ERG has played a key role in orchestrating opposition to Theresa May’s Withdrawal Agreement and has been instrumental in voting it down in Parliament [12,59].

Brexit has therefore in large part been motivated and continues to be significantly influenced by the right wing of the governing Conservative Party [54]. Nick Clegg, the leader of the Liberal Democrats and the Deputy Prime Minister during the 2010–2015 Coalition Government, has written:

I occupied a ringside seat as the Conservatives lurched rightwards under the pressure of an insurgent UK Independence Party (UKIP) and reopened their festering disagreement over Europe, culminating in Cameron’s spectacularly misjudged referendum on the UK’s membership of the EU.

(p. 2) [54]

Arguably, it is difficult to interpret much of the political context of Brexit to be positive for animal protection. Neumayer has examined the positions of parties on the left–right political spectrum and their stances on environmental policy in party manifestos. He found that left-wing parties have more pro-environmental policies than those on the right. His research on the political orientation of individuals corroborated this [60].

The political right is associated with a small state, deregulation and support for business and industry interests. In terms of the state and regulation, Radford writes the following with respect to animal protection:

Because legislation does not in the main protect an animal’s life, the owner retains complete discretion to decide for himself whether it should live or die. Legal regulation of the way in which animals are treated therefore continues to be essential in order to offset the otherwise unconstrained property rights of the owner under common law.

(p. 102) [61]

A political philosophy that favours a small state, deregulation and support for business and industry interests is generally not associated with progressive animal protection policies. In the UK context, this can be seen from animal protection policies of political parties in general election manifestos. For instance, in the UK 2017 general election, the Green Party has far more progressive policy positions than the Labour Party, which in turn has a more progressive stance compared to the Conservative Party [62]. This line of decreasing protection for non-human animals follows the political spectrum from left to right.

Human society has almost absolute power over farmed, experimental and companion animals [63]. Such animals are almost invariably harmed in the farming or experimental process, often severely. The great majority of farmed and experimental animals are killed before their natural deaths [64,65]. Non-human animals are in an extremely weak position and are effectively at the mercy of a Government voted for by humans with a mandate overwhelmingly to promote human wellbeing [66]. The state is the obvious protector of the weakest in society, whether they are elderly, the young or the mentally incapacitated. 

Smaller states with lower rates of taxation and public spending are associated with self regulation and reduced capacity for enforcement of laws. A political philosophy supporting a small state claims that markets distribute goods most efficiently [60]. Thus, right-wing politics supports minimal regulation, deregulation or industry self-regulation. Since sentient animals, like other vulnerable groups in society, require effective laws and their enforcement for protection, such a politics is arguably not optimal for their welfare. On the morality of the market, Radford writes:

If the market has a morality at all, it is not necessarily that of the community, and across a broad spectrum of interests it is considered both legitimate and appropriate to use legislation to offset the extremes of market forces, especially to protect the vulnerable.

(p. 109) [61]

Radford’s claim is particularly valid because animals are classified legally as property and traded in the market as commodities [67,68]. Indeed, it was this reality that motivated Britain, with a history of animal protection, to lobby the EU to recognise animal sentience in law.

The Conservative Government’s approach to animal protection can be illustrated by its policy on animal welfare codes in 2016. In the UK, animal welfare codes are official documents that are not themselves legally binding, but they can be used in a court of law to support a case prosecuting cruelty [61]. They are published for most farmed species and have been considered important documents for guidance for farmers and other animal keepers [69,70,71,72]. However, as a result of chronic underfunding at Defra, the animal welfare codes were desperately in need of being updated. Rather than update the codes in central Government, or commission experts in animal welfare to update the codes, the Conservative Government planned to repeal the codes and replace them with guidance written by the farming industry itself. 

The Conservative Government’s approach to the codes is entirely consistent with the approach to policy making discussed above. The centre right Conservative Government that believed in a small state followed a self-regulatory agenda that supported industry interests to the extent that it asked the farming industry itself to write up-to-date guidance on animal welfare. Predictably, animal protection NGOs and the general public objected to the farming industry, which was seen to have obvious vested interests, updating the documents [73]. The campaign against the policy was successful and the Government was forced to u-turn on its earlier position [74,75].

There is therefore a real concern that both the motivation and delivery of Brexit by the political right will be detrimental for animal protection, based on the above discussion.

### 4.4. Animal Protection Policies of the Governing Conservative Party during Brexit

When discussing how Brexit is likely to impact animal protection, a key indicator is the past record of the Government that is in power and that will deliver Brexit. For instance, it is reasonable to predict that a Government that had a very good record on animal protection issues might be more concerned about how Brexit impacts animals, and would mitigate any threats. Conversely, a Government that had a poor record on animal protection might be less concerned about how Brexit impacts animals. Ultimately, the claim is based on the premise that a more progressive Government on animal protection would have made policies based in part on a higher regard for animal protection. Of course, this relation between a Government’s past record on animal protection and its future policy positions is not a necessary one. I.e. the relation between past record and future policy making would not hold in all instances. Despite this, it is reasonable to claim there is some relation between the record of a Government and future policy positions. In this context, the Conservative Government has a mixed record on animal protection policy, which is summarised in Table 2.

#### 4.4.1. Controversial Animal Protection Policies: Fox Hunting and Badger Culling

The Conservative Government’s approach to two policy issues, first animal sentience, and secondly animal welfare codes, have been discussed above. Further examples include the Conservative Party policy on fox hunting and the Conservative Government’s support for badger culling.

After more than a century long debate, a Labour Government passed the 2004 Hunting Act to outlaw fox hunting. The Hunting Act was passed based on a free vote in Parliament, and it enjoys widespread public support; 84 per cent in a British poll responded that fox hunting should not be made legal [76]. Despite this, the Conservative Party’s 2017 general election manifesto included a pledge to re-open the fox hunting debate by giving a free vote in Parliament to repeal the Hunting Act [77]. Furthermore, the Prime Minister Theresa May has publicly supported a return to fox hunting during the 2017 general election campaign. In his analysis of Brexit and animal protection, Wookey has been severely critical of May in this context, claiming that her support for fox hunting means that she is in ‘no credible position to comment on animal welfare concerns at all’ (p. 41) [35].

The manifesto pledge and May’s support for fox hunting were severely criticised even in Conservative supporting media. Some blamed the fox hunting pledge for losing key votes that resulted in the Conservatives losing seats at the election and forming a minority Government with the Democratic Ulster Party (DUP) [78]. The Conservatives subsequently dropped the policy [79], presumably because it would not command a majority in Parliament to enable its passage. Fox hunting is supported by elements of the Conservative Party based in part on a libertarian politics of not interfering with minority interests [80,81,82].

The 2010–2015 Conservative-led coalition Government and the 2015–2018 Conservative Governments have maintained strong badger culling policies since the pilot culls began in 2013. In the UK, the badger, *Meles meles*, is culled as a disease control measure to reduce bovine tuberculosis in cattle [83]. The National Farmers Union (NFU) strongly supports badger culling [84], and the Conservative Party is considered to be close to the NFU and farming interests [85]. The Government culling policy is despite independent scientists, based on a decade long government-commissioned field trial, recommending against culling. The Independent Scientific Group (ISG), which conducted the Randomised Badger Culling Trial (RBCT) advised that badger culling can make ‘no meaningful contribution’ to reducing bovine TB in cattle [86]. In the UK, the badger is a protected species under the Protection of Badgers Act 1992. It is the largest land carnivore in the UK and is an important wildlife species in British literature and culture. The Conservative Party’s stance on badger culling in Government has, therefore, been highly controversial. Based on Government figures, the policy involves culling five (4.8) badgers to prevent the slaughter of a single cow over a nine year policy timeframe [87,88].

In contrast to the Conservative Government’s badger culling policy, the Labour Party both in Government and in opposition has maintained a strong policy against culling. Based on the report of the ISG in 2007, the Defra Secretary Hilary Benn announced in Parliament that Labour would not pursue a badger culling policy [83]. More recently, the Labour Party manifesto for the 2017 general election states unequivocally ‘We will cease the badger cull, which spreads bovine TB’ [89]. The Labour Party repeated the policy to ‘End the badger cull’ in its 2018 50-point animal welfare plan [90]. Similarly, the Green Party strongly opposes the badger cull and its sole MP Caroline Lucas is a fierce critic of the Conservative policy [91].

Again, the Conservative Government position on badger culling may be instructive for Brexit and animal protection. Arguably, the Conservatives have prioritised the economic interests of the farming industry over the protection of a native wildlife species. This may in part be explained by the power of agricultural policy communities and their influence on government [50,92,93]. However, as stated above the Labour Party has a strong policy against culling and has committed to stop the cull. This may be evidence of the agricultural policy community opening up to competing interests, at least in the case of a Labour Government. An alternative explanation to policy network theory would be that in bovine TB policy Labour has followed an evidence-based policy making model [94] more so than have the Conservatives. Of course, this assumes that the Labour Party would in fact stop the badger cull if it were elected to government.

The Conservative Party policy of widespread culling has been conducted in the face of scientific and public opposition. Brexit will present major conflicts for the government between promoting economic interests, for instance in trade deals, and protecting animal welfare. The badger culling policy may be instructive for how the Conservative Government will act in this context.

#### 4.4.2. Progressive Animal Protection Policies: Mandatory Closed-Circuit Television (CCTV) in Abattoirs, Prohibition of Microbead Plastics and Sale of Ivory, and Lucy’s Law

The controversial policies discussed above and media coverage of issues such as chlorinated chicken have prompted the Conservative Government to make a number of policy statements about maintaining and even improving animal protection during Brexit. For instance, the Prime Minister Theresa May made the following statement during Prime Ministers Questions in reply to a question by the Conservative Brexiteer Theresa Villiers MP:

We should be proud that in the UK we have some of the highest animal welfare standards in the world—indeed, one of the highest scores for animal protection in the world. Leaving the EU will not change that. I can assure her that we are committed to maintaining and, where possible, improving standards of welfare in the UK, while ensuring of course that our industry is not put at a competitive disadvantage.

[95]

The Conservative Government has made a number of important reforms in animal protection. These include mandatory CCTV in abattoirs in England, the banning of microbead plastics, the prohibition of the sale of ivory and Lucy’s Law, which bans the third party sale of puppies. The Conservative Government should be applauded for such policies. Indeed, in 2018 the RSPCA awarded Michael Gove the ‘Politician of the Year’ for his animal protection policies as Defra Secretary [96]. 

Whilst welcome, however, the benefits of the above reforms are relatively small compared to some of the threats that Brexit poses to animal protection. For this reason, it is of fundamental importance for the animal protection community to maintain a critical stance during Brexit [35]. Brexit is a major political upheaval and the risks to animal welfare are substantial. If Government policy and trade agreements permit the import of lower welfare foods, for example, the positive impact of such reforms would be wiped out. This is because the import of lower welfare products would have widespread and long term impacts on animal welfare, which would substantially outweigh any of the benefits based on the above policies. Indeed, the impact these reforms will have on animal welfare is a drop in the ocean compared to larger scale changes in food and farming policy that could have massive detrimentals impact on animal welfare.

### 4.5. Major Time Constraints

Once Theresa May had given the EU Commission notification of Article 50 in March 2017 to leave the EU, the clock was ticking for Brexit. The UK would have only two years to conclude a divorce agreement with the EU until March 2019 before it entered the 20-month implementation period until 2021. Indeed, Robert Peston has called May’s decision to invoke Article 50 ‘the most wilful act of vandalism by a serving prime minister’ (p. 18) [7]. This is because triggering Article 50 created an arbitrary hard deadline and at the same time gave up the UK Government’s most important leverage over the EU. This section briefly discusses two major threats to animal protection associated with time constraints to prepare for Brexit.

#### 4.5.1. Live Animal Transport

Jo Moran of the Brussels-based animal protection NGO Eurogroup for Animals has given a presentation to the European Parliament that highlighted the grave consequences of a hard Brexit on the transport of live animals [97]. The EU and Turkey share a customs union but this does not cover agricultural products. This means there are mandatory health checks on livestock animals being transported between the EU and Turkey. These mandatory health checks can cause six-hour hold ups at the Bulgaria-Turkey border; in some cases the delays are much longer than six hours. To put this in context, Compassion in World Farming (CIWF) lobby for a *maximum* total journey time of eight hours [98]. The reason is that transportation causes live animals substantial stress and suffering, which increase with longer journey times [99].

In the context of Brexit there is a major risk that the Turkey-Bulgaria border situation is repeated at the Dover-Calais sea border between the UK and France. Indeed, the problem could be far worse. The UK Government’s policy is for agricultural products, including livestock, to be part of a ‘common rulebook’, which effectively means they remain in the single market and would not require additional health checks [10,11]. At the time of writing, however, Theresa May’s Withdrawal Agreement has been rejected by Parliament, so it is uncertain if there will be a ‘common rulebook’ for agricultural products. If, for whatever reason, livestock are not permitted free movement of travel between the UK and EU, the consequences for animal protection could be grave.

Modelling studies have shown huge tailbacks on the M20 in Kent in the UK, which is the main artery road that lorries will carry live animals to Dover and on to Europe [100]. Based on current timeframes related to Article 50 and leaving the EU, the UK simply does not have the time to build infrastructure to meet demands for border checks of live animals at Dover and Calais. The problem would arise in the event of a no-deal on Brexit between the UK and the EU, which remains a real possibility as it is the default position without a deal [101]. In its guidance on what to do in a no deal situation, the Government has stated that it will not conduct mandatory health checks on livestock coming from the EU. In an article discussing how a no-deal Brexit might impact food security, Ian Dunt has criticised the Government position on this. First, the situation does not seem consistent with Brexiteer demands to take back control, when we are relying on EU health standards. Secondly, the plan also relies on the EU reciprocating, i.e., not checking live animals being imported from the UK, which is far from certain [102].

#### 4.5.2. Trade Negotiators

A further example of time- and resource-related threats relates to expert trade negotiators. The EU has negotiated on behalf of the UK in trade agreements for the past few decades. Indeed, the EU has a reputation for having some of the best trade negotiators in the world. Free trade deals generally take around seven years to conclude. However, under the Withdrawal Agreement the UK would only have 21 months to make a trade deal with the EU between 29 March 2019 and 31 December 2020. When the UK leaves the EU, it will need professional and skilled trade negotiators to broker deals with the EU and non-EU countries and trade blocs. However, the UK is in the position of having very few trained and experienced trade negotiators [103]. Indeed, Defra has been recruiting negotiators at all levels to work on trade deals after Brexit.

The UK’s lack of trade negotiators is a major problem for animal protection. As discussed in a later section of this paper, a major threat that Brexit poses to animal protection is the import of lower welfare agricultural products. The UK Government has made numerous policy statements to the effect that animal welfare will be maintained after Brexit [95,104,105]. Given that a major threat to animal protection is the import of lower welfare products, the Government negotiating stance for all trade deals must be that animal welfare standards constitute a red line. I.e. the Government position must be that the UK will not import lower welfare animal products. If the UK Government does not commit to and maintain such red lines on importing lower welfare products, it is difficult to see how Brexit will not be detrimental to animal welfare, all things considered.

## 5. Regulatory Changes

### The UK Agriculture Bill

McCulloch [3] claimed that whether leaving the EU benefits or harms animal protection overall will be determined by the impact of Brexit on farmed animals. One reason for this is that there are far more farmed animals compared to other categories of animals used by humans. One billion land farmed animals are raised and slaughtered in the UK each year [22]. This figure compares, for instance, with around four million experimental procedures conducted on animals in the UK annually [23].

The second reason that the impact of Brexit on farmed animals will almost certainly determine its impact on animal protection per se is that leaving the EU will mean greater regulatory changes for farmed animals compared to other categories of animals. Farmed animals are traded as commodities in the EU and across the globe. The EU has competence over large areas of agricultural policy and aims to create a level playing field for farmers across the member states [106,107]. All forms of Brexit mean that the UK will leave the Common Agricultural Policy (CAP) of the EU. For this reason, the UK Government has published a white paper *Health and Harmony: The future for food, farming and the environment in a Green Brexit* outlining its future vision for farming [108]. In the white paper, the Defra Secretary Michael Gove is critical of the CAP:

For more than forty years, the EU’s Common Agricultural Policy (CAP) has decided how we farm our land, the food we grow and rear and the state of the natural environment. Over that period, the environment has deteriorated, productivity has been held back and public health has been compromised. Now we are leaving the EU we can design a more rational, and sensitive agriculture policy which promotes environmental enhancement, supports profitable food production and contributes to a healthier society.

(p. 5) [108]

The Government consulted on the white paper and ultimately published its Agriculture Bill in September 2018 [109]. The Bill provides for the authorisation of agricultural payments in a transition period between 2021–2027 when the UK leaves the EU and CAP. There are two key points to briefly discuss that relate to this paper. First, there is significant potential in the Bill to improve animal protection in the UK. The Bill aims to shift payment of agricultural subsidies away from being based on land area to payment for public goods. Given that the Bill lists animal welfare as a public good, there is clear potential for animal protection to be substantially improved in the UK based on rewarding farmers for high animal welfare standards.

The point above about rewarding high animal welfare standards is important but further discussion is outside of the scope of this paper. This paper is concerned not with the opportunities presented by Brexit, but with the threats posed by it. Hence, a full consideration of the potential opportunities the Bill provides for animal protection in the UK must be left for further research.

A threat to animal protection related to the Agriculture Bill is that it does not contain provision to prevent the import of animal products that do not meet animal welfare standards in the UK. The RSPCA has noted in its response to the consultation on the Bill that the Government rejected an amendment to the Trade Bill in June 2018 to prevent the import of lower welfare products [110]. It recommends that Clause 27 of the Agriculture Bill is amended such that improvements in UK farm animal welfare standards do not mean that British farmers will have a competitive disadvantage compared to, for example, US farmers producing animal products with far less stringent regulations for animal welfare, the environment and food safety. The RSPCA is effectively lobbying Government to make a statement in law to protect British farmers that produce higher welfare products from the risk of importing lower welfare products when the UK leaves the EU.

Finally, the Government has committed to maintaining the level of subsidies as under CAP until 2022 or the end of Parliament. If Government were to reduce overall subsidy levels after this time it may have a negative impact on animal welfare.

## 6. Economic and Trade Factors

### 6.1. Brexit and Economic Impacts on Animal Welfare

Independent economic analyses by the International Monetary Fund (IMF) [111] and the UK Government civil service [112,113] have forecast that Brexit will negatively impact the economy in the medium term. The UK Treasury [114] and the Bank of England [115] have published forecasts in late 2018. The UK Treasury analysis finds that the three Brexit models it analysed would lead to a reduction in gross domestic product (GDP). A Norwegian model (the UK joining the European Economic Area) would lead to a fall of 3.8%; a Canada free trade model would lead to a fall of 6.2%; and a WTO rules Brexit would lead to a fall of 7.5%. To put these figures in perspective, the 6.2% reduction in GDP in the Canada model translates to a fall of £4300 per household. The London School of Economics (LSE) Centre for Economic Performance has claimed that the Treasury has likely underestimated the cost of Brexit and that the long-term impacts on the UK are likely to be even higher [116]. The LSE group summarise the Treasury report as follows:

And the Report is essentially saying that whatever unexpected events happen in the world, the UK is likely be considerably poorer than it would have been if it remained in the EU.

(p. 7) [116]

The Bank of England report predicts that a ‘disorderly’ no deal Brexit could cause a fall in GDP by up to 8% in one year alone and for unemployment to rise by 7.5%. A ‘disruptive’ Brexit, where the UK maintained some trade agreements, could lead to a reduction in GDP of 3% over five years [115,117]. Hence, both UK Treasury and Bank of England reports, in line with the IMF and Government civil service reports, predict that Brexit will negatively impact the UK economy.

Analyses have not been conducted on how the negative impact on the UK economy will impact animal protection. Despite this, we can be confident that the negative economic impact of Brexit on the wider UK economy will have a detrimental effect on animal welfare. This is because negative economic indicators are generally associated with a negative impact on animal welfare. There are a number of mechanisms by which this happens at the level of the producer, consumer, citizen and government. For instance, farms that are struggling economically are often associated with poor welfare [118]. Grant has claimed that Brexit will pose a series of challenges to UK farmers and writes ‘it is difficult to see that [the consequences] would, on balance, be advantageous’ (p. 16) [119]. Livestock farming is based on tight profit margins and economic changes as a result of Brexit will cause some farms serious difficulties that will impact animal welfare. Secondly, reduced disposable income for British consumers, and indeed EU27 consumers affected by Brexit, will translate to reduced purchasing power to buy higher welfare products at supermarket checkouts [120]. Thirdly, reduced disposable income of pet owners translates to spending less on companion pet dogs, cats and other species. The *Guardian* headline ‘Dumped pets pay price of recession’ reported that 57% more pet animals were abandoned in 2008 compared to 2007, when the recession started [121]. This headline illustrates the impact of a recession on companion animals. Fourthly, reduced disposable income risks reduced charitable donations to animal protection organisations. Indeed, the RSPCA fell from seventh to tenth place in the most popular fundraising charities between 2007–2010 [122].

At government level, reduced Treasury tax receipts can lead to budgetary cuts that impact animal welfare in myriad ways. The austerity policy of the 2010–2015 coalition and 2015–2017 Conservative Governments has meant massive cuts to Defra [123]. Defra has suffered the largest budget cuts of any UK Government department [124]. The *Farmers Guardian* has reported that the Defra budget has been cut by around a half between 2008 and 2017 [125]. Enforcement of animal protection, which is poorly resourced at the best of times [126], has suffered. Codes of recommendations for livestock had not been kept up to date [127]. The Conservative Government’s proposal to replace these with industry-based guidance and its subsequent u-turn due to public disquiet has been discussed earlier in this paper.

In summary, when there is a reduction in the wealth of human society, there is a knock-on effect on animal protection. Indeed, it is often claimed that economic downturns impact those on lower incomes in society more than those at the top. Given that animals are generally considered to be at the bottom of society, if indeed they are considered to be part of society at all [128], we can reasonably expect economic downturns to have a disproportionate impact on animals.

### 6.2. A Major Threat of Brexit: Importing Lower Animal Welfare Products

Arguably, the major immediate threat Brexit poses to animal protection is the import of meat, dairy and other agricultural products that have been raised in standards far lower than those mandated by law in the UK and EU. McCulloch [3] has discussed the UK farm animal welfare regulatory context as a member state of the EU. High tariffs, regulations on slaughter and various binding agreements largely prevent the import of lower welfare products. In its 2017 report *Brexit: Farm Animal Welfare*, the House of Lords committee conclude that:

Our evidence strongly suggests that the greatest threat to farm animal welfare standards post-Brexit would come from UK farmers competing against cheap, imported food from countries that produce to lower standards than the UK. Unless consumers are willing to pay for higher welfare products, UK farmers could become uncompetitive and welfare standards in the UK could come under pressure.

(p. 18) [106]

In cases where the UK does not conclude trade deals, trade is based on World Trade Organisation (WTO) rules. The WTO is an organisation that promotes free trade between nations and aims to prevent protectionist policies [129]. If a WTO member believes any other member is using tariffs or other instruments to illegitimately protect its own industries, it can challenge this situation at the WTO. 

Higher welfare standards often have a greater financial cost to farmers compared to lower welfare standards. Hence, governments with higher welfare standards, such as the UK, aim to protect their industries on the grounds that it costs their farmers more to produce meat and dairy products as the regulations in their country are more stringent [130]. WTO rules do provide a number of exceptions to its prohibition on trade restrictions. One of these is for measures necessary to protect public morals, and the WTO has recognised that animal welfare falls within the scope of public morals [131]. However, whether challenges made at the WTO to trade restrictions based on animal welfare would be upheld remains highly uncertain [1,106].

### 6.3. Policy Measures to Prevent a Race to the Bottom

The House of Lords European Union Committee has stated that it ‘encourages the Government to secure the inclusion of high farm animal welfare standards in any free trade agreements it negotiates after Brexit’ (p. 78) [132]. What policy measures can be used to mitigate the import of lower animal welfare products and a race to the bottom? Stevenson [131] addresses this question in his report *A Better Brexit for Farm Animals: What the government must do to protect welfare standards*. He proposes that the Government insist on a clause in trade agreements permitting the UK to require imports to meet UK farm animal welfare standards. Stevenson also proposes that Parliament pass legislation to ensure that Government maintains this position. Furthermore, he argues that Parliament should have a decisive role setting goals in trade agreements, monitoring their negotiation at regular intervals, and ratifying their conclusions [131]. 

Stevenson is effectively suggesting that Parliament vote to bind the Government’s hands on animal welfare when negotiating trade deals. If the Government is genuinely committed to maintaining animal welfare standards, this should not be a problem. However, despite Stevenson’s reasonable proposal, it seems incredibly unlikely that Parliament would do this. Prior to the summer recess after the 2017–2018 Parliament there were major debates about Parliament having a meaningful vote on the final Brexit terms [133,134]. The Government’s argument, supported by many in Parliament, and especially Brexit-supporting MPs on both sides of the House, was that a meaningful vote would undermine the Government’s negotiating position.

Returning to trade deals, Dunt has reported how under the Ponsonby Rule Parliament has the right to object to the ratification of trade deals. Parliament has 21 days to object, but since the Government can repeatedly put forward the same trade deal, Dunt describes the Ponsonby Rule as a ‘pretty weak’ parliamentary power. Indeed, Dunt is highly critical of the Government position when debating the Taxation (Cross-Border Trade) Bill, in his aptly titled article ‘Dirty tricks: Trade deal legislation strips parliament of control’ [135]. Brexit has revealed a theme of how Government has limited the power of Parliament. Indeed, MPs voted by 311 to 293 to find Government in contempt of Parliament in December 2018 for failing to disclose its legal advice on Brexit [136]. This is problematic for animal protection, since Parliament is arguably more progressive in this policy area than Government.

Stevenson further recommends that the Taxation (Cross-border Trade) Bill be amended, which is now an Act of Parliament. The Bill set out factors the Treasury must have regard to when setting tariff rates for imports. Stevenson proposed that the Bill should be amended such that the Treasury must have regard for (i) the interests of the farming industry, and (ii) maintaining UK standards of animal welfare. Stevenson had reported that the Department for International Trade was opposed to such amendments, which ‘casts some doubt on the strength of the Government’s commitment to safeguarding UK animal welfare standards’ (p. 10) [131]. Indeed, the Bill was subsequently amended to include ‘the interests of producers’, but a proposed amendment to maintain UK standards of animal welfare was rejected by the Government. The inclusion of reference to the interests of producers may ultimately benefit animals. However, the Conservative Government’s explicit recognition of the interests of the farming industry and its rejection of the explicit recognition of farmed animals in the Taxation (Cross-border Trade) Act arguably represents a further example of promoting industry interests and the denial of nonhuman interests.

The effect of Parliament abrogating responsibility to ratify free trade agreements and of the reluctance of the Conservative Government to include animal welfare in the Taxation (Cross-border Trade) Act should be clear. Brexit poses serious threats to welfare in the form of importing animal products raised to far lower standards. The threat has the potential to impact the welfare of billions of animals going forward. The risk that this threat materialises seems substantially higher if the Government of the day has free rein to judge how important animal welfare is in multi-billion dollar trade agreements. 

Liam Fox’s Department for International Trade (DIT) has since published consultations on future trade with the US, Australia, New Zealand and access to the Comprehensive and Progressive Agreement for Trans-Pacific Partnership (CPTPP) [137]. The Department published information packs that accompany the consultations for these bilateral free trade agreements. The contents include a section entitled ‘Impacts: How different groups might be affected by a Free Trade Agreement’. The contents then list separate sections for potential impacts on businesses, consumers, workers, wider social impacts and the environment [138]. 

Despite the major concerns raised by Stevenson and many published Brexit and animal protection reports about the importance of maintaining animal welfare standards in trade deals, the impact on farmed animals in the DIT documents is notable by its absence. There is no reference to the many billions of farmed animals even under the wider social impacts or environment sections. Again, this is a very big concern for animal protection post-Brexit. The Department of International Trade, which will have substantial influence on trade deals, has effectively excluded farmed animals in its consultation documents as a group that will be impacted. This is despite the real possibility that the biggest impacts of Brexit may well not be on humans, but on farmed animals [139]. It is also despite the fact that there has been widespread media coverage of the potential impacts of post-Brexit trade on food security and animal protection, for instance in the form of concern about chlorinated chicken [140,141,142].

The political situation leaves a small number of individuals in the UK Cabinet with substantial influence over the welfare of billions of farmed animals, largely unchecked by Parliament. This is worrying. A recent exposé appears to show that the Institute for Economic Affairs (IEA), a right-wing government think tank close to the Conservative Party, was taking cash for access to senior ministers. In the video footage, a Greenpeace undercover operative posed as a representative of US agricultural interests, and was offered significant access to Conservative Government ministers such as the Brexiteer Defra Secretary Michael Gove [143]. Prior to this incident, Peter Stevenson had warned of precisely this kind of situation, and its implications for animal welfare, in his report:

Influential voices argue in favour of diluting UK standards in order to facilitate trade deals, unilaterally removing import tariffs and ending farm subsidies. Such moves would lead to UK farmers being undermined by lower welfare imports. If the UK cannot protect them from such imports, farmers may, understandably, resist welfare improvements and may even press for existing welfare standards to be lowered.

[131]

The following section moves on to discuss risks to animal protection based on the loss of access to EU institutions and other capacity to safeguard animal welfare.

## 7. Institutional and Capacity-Related Factors

### Loss of Advisory Bodies and Enforcement Institutions

Brexit means the loss of EU institutional capacity that supports animal protection. The Trade Control and Expert System (TRACES) tracks the live movement of animals into and within the EU. The TRACES system also underpins the Tripartite Agreement between the UK, France and Ireland on the movement of equine animals. When it leaves the EU, the UK will need to establish its own system to trace live animals [97,144]. The UK Import Control System is now taking over this role to trace live animals [145].

EU member states are members of the European Chemicals Agency (ECHA) and the EU Reference Laboratory for Alternatives to Animal Testing (EUL-ECVAM). The ECHA authorises market access to chemicals used in the EU. The ECHA provides a foundation for the Registration, Evaluation, Authorisation and Restriction of Chemicals (REACH) legislation. Collectively, these agencies share data tested on animals and avoid the duplication of unnecessary animal experiments. A major threat of Brexit is that it will lead to the unnecessary testing on animals as a result of the UK leaving these institutions [144].

Finally, the European Food Safety Authority (EFSA) has a role to inform the EU Commission on animal health and welfare. It is a highly respected body of expert scientists that has produced a number of helpful reports on animal welfare issues [99,146]. Brexit will mean that that the UK loses access to EFSA.

## 8. EU and International Factors

### 8.1. Common Agricultural Policy (CAP) Funding

The Common Agricultural Policy (CAP) serves to increase productivity, ensure a fair standard of living for farmers, stabilise markets and ensure food security [107]. The CAP accounts for around 39% of the overall EU budget [4]. Around half of farm income is based on CAP payments. The UK is a net contributor to the CAP. There is therefore a risk that Brexit will result in a reduction of the CAP budget. For instance, Matthews has stated:

Because the UK is a net contributor to the EU budget, a net importer of agri-food products from the EU, and punches above its weight in research terms, its withdrawal would have broadly negative effects for the EU farm and food sector.

[147]

As farmers rely on a substantial proportion of income from the CAP, any reduction may have a detrimental impact on farm animal welfare in the EU. In his presentation to the European Parliament the Political Adviser to the NGO EuroGroup for Animals Jo Moran has claimed that the withdrawal of the UK from the EU may over time have a significant impact on animal protection as a result of a reduced CAP budget [97].

### 8.2. Brexit Means Animal Welfare Will Be Weakened in the European Union

The UK has a history of being a regional and global leader in animal protection [61]. Moran has described the UK as a ‘beacon for animal welfare’ in the EU [97]. As an independent nation, the UK can improve its animal welfare standards within its own borders. In a globalised world, a nation with progressive animal protection policies can influence other nations through free trade agreements. In the EU, Germany, France and the UK are the larger and more politically powerful nations [148]. As a member of the EU, the UK has leveraged its political and economic power to positively influence animal protection in the remaining 27 member states of the Union [4]. Thus, the UK, in concert with some other EU member states, has had a very substantial positive influence on a much larger market of 510 million citizens and consumers than it could have done outside of the EU [97]. Given this, there is a major risk that the political will for progressive animal protection reforms will be diluted when the UK leaves the EU. Indeed, given the status of the UK as a political heavyweight in the EU as well as a leader in animal protection, it seems difficult to see how the political will for animal protection reform within the EU will not be weakened after Brexit. The animal protection community will need to hope that other progressive nations in the EU such as Sweden, Germany, the Netherlands and Denmark maintain current political pressure without the UK as an EU ally.

### 8.3. Weakened Animal Welfare Lobby in European Union Impacts Animal Protection Internationally

As the UK has leveraged its economic and political power to influence the EU, the EU has leveraged its economic and political power to influence animal protection internationally [149]. The EU largely prevents the import of lower welfare products based on tariff and non-tariff barriers. If third countries wish to access the large EU market, they must at least match EU farm animal slaughter standards. Furthermore, the EU has maintained strong positions against the US on not importing chlorinated chicken and hormone beef. In trade deals, the EU has had a more direct influence on animal welfare standards internationally. For instance, Chile now recognises animals as sentient based on the EU–Chile trade agreement. The progressive nature of animal protection in the EU has likely influenced reform in US states such as California, Michigan and Ohio to prohibit intensive farming practices such as barren battery cages, calf crates and sow stalls.

Hence, if Brexit weakens EU reforms on animal protection, this will not only impact animals in the EU, but potentially internationally. The impacts of Brexit on the EU and internationally are impossible to quantify with any degree of precision. Despite this, it seems reasonable to claim that there will be a significant negative impact, as the UK has to date leveraged its influence through the EU. Many have argued that Brexit will leave both the EU and the UK politically and economically weaker [54,103]. The same reasoning can apply to the political influence of both the UK and the EU in the specific area of animal protection. The problem is, of course, that the numbers of animals affected at the EU and international level is very substantial. Whereas the UK raises and slaughters one billion land farmed animals annually, the figure for the EU is 4.7 billion and for the US, for instance, is 10 billion [3]. Hence, any negative impact at the international level that results from Brexit weakening the total political and economic influence of the EU and the UK on animal protection has the potential to have very detrimental impacts. Indeed, it is not unreasonable to claim that if there is such a detrimental impact at the international level, it will outweigh all other impacts, simply by virtue of the vast number of animals that are affected.

## 9. Conclusions

The British people voted in a 2016 referendum to leave the EU. The UK has been a member of the EU since 1993 and before that a member of the EC since 1973. EU laws have had a substantial impact on UK animal protection policy. Additionally, the stance of the UK Government on animal health and welfare has had a major impact on EU policy. Brexit therefore has great potential to impact animal protection both negatively and positively, in the UK, EU and internationally.

A number of reports have been published on the threats and opportunities that Brexit presents for animal protection. Whether these threats and opportunities materialise will be determined by a range of factors including the decisions of political actors in Westminster, Whitehall, Brussels and further afield. Additionally, the interpretation of WTO trade rules on restrictions for animal welfare will influence how Brexit affects animal protection. This paper has assessed how animal protection might be impacted given the political context of Brexit, using the EU/UK regulatory relationship as a baseline. Specifically, it discusses the threats that Brexit poses to animal protection. The paper discusses threats to animal protection in terms of five criteria. These are first, political context; second, regulatory changes; third, economic and trade factors; fourth, institutional and capacity-related factors; and fifth, EU and international considerations.

A major threat to animal protection is the import of agricultural goods produced in lower animal welfare conditions. This would undermine UK farmers who are likely to oppose welfare improvements and indeed may well press the Government to lower existing welfare standards to enable them to compete with imports on a level playing field. The EU has the most progressive animal welfare laws in the world. The US, a large exporter and potential trade partner, has some of the lowest standards in the developed world. If the UK Government were to permit the import of lower welfare products, it is difficult to see how Brexit could have a net positive impact for animals. A proportion of British consumers would purchase, for example, US produced chicken, eggs or beef, which would mean a shift towards the consumption of lower welfare products. The import of cheaper lower welfare products would disincentivise British farmers from improving animal welfare going forwards, and they may even lobby Government to relax current UK regulations.

The political context is a weak minority Conservative Government with a politically powerful right wing of the Parliamentary Conservative Party supporting a hard Brexit. The political right is associated with a small state, low taxation and deregulation. Many on the right promote free trade through *laissez faire* economics. Farmed animals are legally considered as property and are traded dead or alive as commodities. It seems problematic to claim that a Brexit motivated and delivered by the political right in the current context is likely to reap benefits for the many animals that will be affected by it.

Furthermore, the modern Conservative Party has a mixed relationship with animal protection, evidenced by the recent media furore over animal sentience and the party’s pledge to hold a free vote on fox hunting. Indeed, the Conservative Government’s rejection of an amendment tabled to include the provisions of Article 13 recognising animal sentience in the EU Withdrawal Bill was consistent with the politics of a small state and deregulation. Ultimately, the Conservative Government was forced to publish its own Bill on sentience. However, the duty of the *state* to pay ‘full regard’ to animals has been watered down to *ministers* paying ‘regard’. Given that local government has a substantial role in animal protection, limiting the duty to government ministers reduces its scope in this context. Furthermore, at the time of writing the Government has proposed a further consultation on the Bill. There is a risk that the UK will enter trade negotiations with nations such as the US, without the Government having the firm duty to pay full regard to animal welfare, previously conferred by Article 13 of the Treaty of Lisbon.

A further major threat to animal protection relates to the loss of political influence of the UK in the EU. The UK has leveraged its political and economic power in the EU to improve animal protection in a far larger market of 510 million consumers. Furthermore, this impact has then been multiplied by the impact of the EU at an international level. The political influence of the progressive bloc of the UK, Sweden, Germany and Denmark in the EU will be weakened by Brexit. Despite the UK having a history as a regional and international leader in animal protection, it will be weaker outside of the EU and will be trading alone with far more powerful nations such as the US. Indeed, the loss of political influence of the UK within the EU and its resultant impact on animal protection in the EU and internationally may be the most substantial threat to animal protection. This follows to a significant degree from the far larger numbers of agricultural animals in the EU and internationally compared to those in the UK.

This paper has assessed the threats that Brexit poses to animal protection in the UK, EU and internationally. In the current political context of Brexit, the threats are very real and there is a significant risk that Brexit will, for instance, lead to the import of lower welfare meat and dairy products to the UK. All things considered, will Brexit be positive or negative for animal protection? Brexit certainly poses very real risks to not only British animals but those in the EU and internationally. However, whether Brexit will be positive or negative for animal protection will also depend on an appraisal of the opportunities that Brexit presents. That is a subject for further research.

## Figures and Tables

**Table 1:** Threats posed by Brexit to animal protection.

Factors	Threat	Notes
Political	The EU has the most progressive animal welfare laws in the world	Given that the EU has the most progressive animal welfare laws in the world, it is prima facie problematic to claim that leaving it is likely to be positive for animal welfare.
	Inherent threats in massive political change	The scale and magnitude of governance and policy affected by Brexit entails inherent risks.
	Right-wing nature of Brexit	Politics of the right generally not associated with progressive animal welfare. Small state, deregulation and support for business and industry often conflicts with animal protection.
	Record of governing party during Brexit on animal protection policy	The Conservative Party is generally not considered progressive on animal welfare. Policy examples are 2017 manifesto pledge for free vote on Hunting Act; support for badger culling in face of scientific and public opposition; and industry self-regulation such as repeal of animal welfare codes and replacement with industry guidance.
	Major time constraints	Live animal transport delays at EU–UK border due to insufficient time to build border infrastructure. Insufficient time to train experienced trade negotiators to represent the UK in post-Brexit trade deals. Two-year period after giving notice of Article 50 means time has been against the UK negotiating EU divorce agreement.
Regulatory changes	UK Agriculture Bill	Government has committed to maintaining Common Agricultural Policy (CAP) level of subsidies until 2022 or end of Parliament. If the Government later reduces subsidy levels it may impact animal welfare. Agriculture Bill has no provision to protect British farmers from the import of lower welfare products.
Economic and trade	Risk of Brexit to UK economy	Impact assessments reveal all forms of Brexit have negative impact on UK economy. Economic downturns are inversely associated with progressive animal protection reform.
	Import of cheaper agricultural goods produced to lower animal welfare standards	EU regulation and tariffs act as protective fortress for animal welfare. Post-Brexit UK may reduce tariffs to promote trade. Stringent UK laws mean higher production costs and retail prices. Lower tariffs leads to import of cheaper products raised and slaughtered to lower welfare standards, e.g., from US. This is a major threat of Brexit to animal protection and is a far greater risk in a hard Brexit scenario. The threat will materialise with a WTO rules-based Brexit.
Institutional and capacity-related	Loss of access to advisory bodies and enforcement institutions	Trade Control and Expert System (TRACES) tracks live movement of animals within EU. European Food Safety Authority (EFSA) is expert body that informs EU Commission on animal health and welfare. European Chemicals Agency (ECHA) and EU Reference Laboratory for Alternatives to Animal Testing (EUL-ECVAM) prevent duplication of testing.
EU and international	CAP funding	The UK is a net contributor to CAP. Post-Brexit CAP shortfall would need to be made up by other nations to maintain CAP spending levels. This means possible CAP reductions, which may negatively impact animal welfare in the EU.
	Brexit means animal welfare weakened in EU	The UK has been a beacon for animal welfare in the EU. Given the leading role of the UK, Brexit means reduced political influence and potential for reform for animal protection in the EU.
	Weakened EU impacts animal protection on international basis	A less progressive EU on animal protection means weaker animal protection positions, e.g., when negotiating free trade agreements with third countries. Leads to longer term negative impact on animal protection internationally.

**Table 2:** Progressive and regressive animal protection policy of the Conservative Government delivering Brexit.

Progressive Policy	Regressive Policy
Mandatory closed-circuit television (CCTV) in abattoirs	Animal sentience policy related to Article 13 of the Treaty of Lisbon
Prohibition of microbead plastics in UK	Policy to repeal animal welfare codes and replace with industry-based guidance
Ban on sale of ivory in UK	2017 general election pledge to give Parliament a free vote to repeal Hunting Act
Lucy’s Law to ban the third-party selling of puppies and kittens	Policy of widespread badger culling in England
